# Parameters of Memory Reconsolidation: Learning Mode Influences Likelihood of Memory Modification

**DOI:** 10.3389/fnbeh.2020.00120

**Published:** 2020-09-24

**Authors:** Katharine C. Simon, Lynn Nadel, Rebecca L. Gómez

**Affiliations:** ^1^Department of Cognitive Sciences, School of Social Sciences, University of California, Irvine, Irvine, CA, United States; ^2^Department of Psychology, College of Science, The University of Arizona, Tucson, AZ, United States

**Keywords:** reconsolidation, learning, memory, learning mode, memory modification

## Abstract

When previously consolidated hippocampally dependent memory traces are reactivated they enter a vulnerable state in which they can be altered with new information, after which they must be re-consolidated in order to restabilize the trace. The existing body of literature on episodic reconsolidation largely focuses on the when and how of successful memory reactivation. What remains poorly understood is how the nature of newly presented information affects the likelihood of a vulnerable episodic memory being altered. We used our episodic memory reconsolidation paradigm to investigate if the intention to encode impacts what subsequently becomes attributed to an older, reactivated memory. Participants learned two lists of objects separated by 48 h. We integrated a modified item-list directed-forgetting paradigm into the encoding of the second object list by cueing participants to learn some of the objects intentionally (intentional learning), while other objects were presented without a cue (incidental learning). Under conditions of memory reactivation, subjects showed equal rates of memory modification for intentionally- and incidentally-learned objects. However, in the absence of reactivation we observed high misattribution rates of incidentally-learned objects. We consider two interpretations of these data, with contrasting implications for understanding the conditions that influence memory malleability, and suggest further work that should help decide between them.

## Introduction

Consider the following: one day in June 2018 you spend a lovely Saturday afternoon playing soccer in a park with a group of friends. The grass is green, the sun is warm, you score a goal, and everyone has a good time. Now imagine that you go back to the same park for another game with the same friends in August, just two months later. On this second visit, there was watermelon and someone flying a kite; but, in other respects, the two afternoons were similar. One year later, how will you remember that first day in the park? We now know there is a good chance that you will remember it as having watermelon. This phenomenon, where memories can be reactivated and modified through updating, is an example of what has been called reconsolidation (Przybyslawski and Sara, 1997; Nader et al., 2000; Sara, 2000).

**FIGURE 1:**
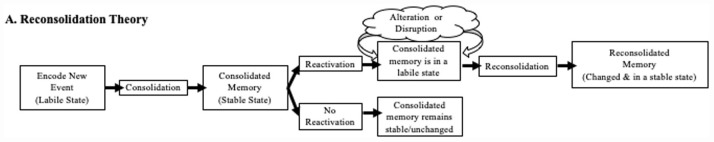
Schematic of reconsolidation theory. New events are encoded, of which the details are consolidated for long-term memory. After consolidation, an older memory can be reactivated and returned to a labile state (top pathway). Once labile, memory traces become susceptible to alteration (strengthening, weakening, or alteration). The memory must proceed through a second phase of consolidation (reconsolidation) to return to a stable memory state. If reactivation does not occur (bottom path), the memory trace remains in a stable state and is not open to direct alteration.

Though it may once have been thought that memory works like a video camera, objectively recording the events we experience, in fact it is more complicated than that (see Figure 1). One forms a memory of the many details of an event, such as friends’ names, the location of the picnic, foods eaten, and games played. The memory for the linkage between these details undergoes a period of stabilization, resulting in the formation of a putatively ‘consolidated’ memory trace (McGaugh, 2000). This trace can later be cued, either externally or internally, causing ‘reactivation’ of that memory. Crucially, absent reactivation the original memory remains stable and intact, unlikely to be altered. In the example above, returning to the same park served as a retrieval cue, reactivating the first “park” memory.

Factors influencing memory trace reactivation include the type of reactivation cue, the relationship between the retrieval context and that of the initial event (in our example the contexts are identical – the same park), the strength of the old memory (Bryant et al., 2019), the timing between the formation and reactivation of a memory (Milekic and Alberini, 2002; Suzuki et al., 2004; Debiec et al., 2006; Morris et al., 2006; Lee, 2009; Wang et al., 2009), the type of memory (Exton-McGuinness et al., 2014), and individual differences (Soeter and Kindt, 2013), including sex (Drexler and Wolf, 2017). Once reactivated, a memory trace appears susceptible to modification. Post-reactivation events, such as behavioral or pharmacological manipulations, can impact the likelihood of reconsolidating an original memory. In fear-based reconsolidation paradigms using classical conditioning, interventions can lead either to the maintenance of the fear response (e.g., freezing), or its attenuation (e.g., loss of freezing behavior), where a new and competing extinction memory is formed (Misanin et al., 1968; Kindt et al., 2009; Monfils et al., 2009; Merlo et al., 2014). Similarly, studies exploring reconsolidation in appetitive learning, such as drug addiction, also frequently use classical conditioning paradigms. Here, post-reactivation pharmacological interventions can block reconsolidation and disrupt the animal’s long-term drug seeking behavior (e.g., Lee et al., 2006). In episodic memory reconsolidation, as in the example of the two park trips, modification of the initial June memory could include (1) the incorporation of new information, defined as intrusions (Milekic and Alberini, 2002; Suzuki et al., 2004; Debiec et al., 2006; Morris et al., 2006; Hupbach et al., 2007, 2009; Lee, 2009; Wang et al., 2009), which in our example could be reflected in remembering (incorrectly) that you ate watermelon, (2) the weakening of some aspects of the reactivated memory (Walker et al., 2003), which might result in subsequently remembering only the gist of the initial experience, (3) the loss of certain details of the initial event (Nader et al., 2000), such as the fact that some friends joined you, or (4) no alteration, in which the memory retains its original details without modification.

After memory reactivation, whether there has been modification or not, the memory must undergo reconsolidation to re-stabilize the trace (Hupbach et al., 2007; Nadel and Sederberg, in press). In episodic reconsolidation, a modified memory trace retains many of its original details, mixed with new details—intrusions— reflecting events that occurred while the original memory was reactivated. Details from the original event rarely if ever intrude into the memory for the new experience that triggered reactivation of the memory for the original event. That is, memory alteration, reflected in intrusions, is unidirectional and only seems to affect the original memory. In our example, details of the August park trip might be attributed to the June park trip, but not the reverse. To date, episodic memory reconsolidation research has largely been limited to questions of when and how older memories are reactivated, and the resulting consequences of that reactivation for original memory details.

More recent research has attempted to understand the neural mechanisms underlying the modification of a reactivated episodic memory. In a recent fMRI study looking at both memory formation and subsequent reactivation, Gershman et al. (2013) trained participants on two lists of objects. They trained List 1 objects with a series of scenes presented between each object while participants were being scanned. For example, participants saw an object (e.g., a picture of a lamp) followed by a series of scenes (e.g., pictures of mountains, forests, and oceans) and then another object (e.g., the picture of a telephone). Days later, participants returned to the same scanner to reactivate the original object memory. Participants were then trained on a new set of objects (List 2) without interpolated scenes to see if neural activation patterns observed during this second list-learning task would predict which objects were subsequently misattributed to List 1. The degree of reinstatement of the neural signatures from List 1 just prior to the presentation of a List 2 object predicted list attribution for individual objects. If scene reinstatement was present, the List 2 object was more likely to be incorrectly recognized as a List 1 object. If scene reinstatement was not present, the List 2 object correctly retained its List 2 identity. Thus, the extent of reinstatement of the brain state associated with List 1 learning played a critical role in determining if a List 2 object would, or would not, be remembered as being on List 1.

We used a similar fMRI paradigm to investigate neural activation patterns associated with memory reactivation and new learning (Simon et al., 2017). Participants also learned two lists of objects; however, for List 1, subjects learned objects and their associated sounds (e.g., participants saw a train and heard a train whistle). After a 2-day delay, we attempted to reactivate participants’ List 1 memory by playing half of the associated sounds from the first list in the scanner. This allowed us to investigate the degree to which the brain reinstated the List 1 object memory prior to new learning. We then taught subjects a second, new list of objects without sounds. First, the degree of reactivation of the original List 1 affected the likelihood of List 1 original memory modification. Greater activity in visual cortex during the sound-induced reactivation of List 1 memory was associated with lower rates of modification of this original memory. This suggests that the more faithfully an old memory is reactivated *before* List 2 learning, the less likely it is that new information is attributed to the initial event (List 1 memory). Second, the amount of temporal parietal junction (TPJ) activity during the encoding of individual List 2 objects predicted whether the initial memory would be updated, or an entirely new memory formed. Specifically, lesser TPJ activation resulted in subsequent mis-attribution of the List 2 object to List 1 (intrusion), whereas greater TPJ activation during the encoding of a specific List 2 object resulted in correct recognition of that item. The TPJ has previously been suggested to play a role in memory-error prediction which impacts reconsolidation to the extent that the degree of difference between the reactivated old memory and what subjects expect and encounter during new List-2 object presentation determines whether they integrate and attribute the new List 2 objects to the initial memory (intrusions), or retain them as a separate, independent memory resulting in correct List-2 recognition (Exton-McGuinness et al., 2015; Simon et al., 2017). Simon et al. (2017) argued that decreased TPJ activation reflects lower prediction error and ultimately greater linkage of the List 2 object to the reactivated List 1 object memory.

Together, these fMRI studies provide some insight into the mechanisms supporting memory modification. First, after Simon et al. (2017), the extent of reactivation of the original memory immediately preceding List 2 learning determines that trace’s susceptibility to alteration such that strong reactivation of the initial memory may better demarcate the List 1 and List 2 learning experiences. Second, after Gershman et al. (2013), the attribution of a new List 2 object can be influenced by the extent of the reinstatement of List 1 contexts immediately prior to that object’s exposure. Third, less TPJ activation at the time of List 2 encoding results in greater attribution of List 2 objects to List 1 (Simon et al., 2017).

What remains relatively unexplored is how the type of new information participants encode during List 2 learning affects the likelihood of a reactivated, vulnerable, episodic memory being altered. We do know that frequency of exposure to new instances contributes to memory alteration. Using a similar reconsolidation paradigm with emotional and neutral pictures, Wichert et al. (2013) demonstrated that the strength of new encoding of List 2 objects influenced subsequent attribution. After reactivating a previously formed picture-List memory (List 1), the authors presented subjects with new pictures either once or three times (List 2). List 2 pictures presented three times had a higher likelihood of being attributed to the old memory than pictures presented once. This demonstrates that subjects must encode new information to a sufficient degree for it to factor into the alteration of a prior memory. Given that factors such as attention, emotion, motivation and learning intention influence the encoding and consolidation of new memories (Rugg et al., 1997; Payne et al., 2008; Aly and Turk-Browne, 2016), we would not be surprised if such factors also influence memory reconsolidation.

In the present study we sought to address an unexplored factor in memory reconsolidation by asking the following question: does learning intention influence the likelihood that new information will be attributed to an older, reactivated memory? We addressed this question using a modified item-method directed-forgetting (IMDF) paradigm and created two distinct List 2 learning strategies within a single subject. During IMDF paradigms, to-be-learned items (e.g., words, pictures, or movie clips) are presented followed by a cue that signals whether each is to be remembered or forgotten (Bjork and Woodward, 1973; MacLeod, 1989; Hourihan et al., 2009; Quinlan et al., 2010). As such, learning intention is quite different between to-be-remembered and to-be-forgotten items. Unsurprisingly, at subsequent testing, memory performance for ‘remember’ items is greater than for ‘forget’ items. This result is thought to stem from a combination of cognitive mechanisms including the intentional deployment of strategies to remember specific cued items and the withdrawal of cognitive processing for others, the intentional inhibition of to-be-forgotten cued information, or both (Taylor, 2005; Fawcett and Taylor, 2010; Taylor and Fawcett, 2011; Fawcett et al., 2013).

The IMDF paradigm creates an opportunity to alter the learning conditions within our reconsolidation paradigm for some of the List 2 objects. In previous research, we incorporated an IMDF paradigm into the viewing of continuous movie clips. Subjects retained gist-like knowledge of the specific content when provided the forget cue versus more accurate knowledge of the specific content when provided the remember cue (Fawcett et al., 2013). Also, Saletin et al. (2011) used an IMDF paradigm to investigate the impact of a nap compared to wakefulness on retention of to-be-remembered and to-be-forgotten words over a 6-h delay, showing that IMDF-cued words can be retained and impacted differentially across delays containing sleep. The embedding of such an IMDF protocol into our typical memory reconsolidation paradigm in List 2 learning allows us to investigate the impact of different learning modalities on subsequent intrusion rates.

In the present study we did not want our participants to intentionally forget. Rather, we wanted to enhance the strength of learning of a specific subset of List 2 objects. We thus provided a “to-be-remembered cue” along with half the objects while the other half were not accompanied by any instruction. Therefore, we had two types of List 2 objects: (1) those to be intentionally learned (presented with a to-be-remembered cue) and (2) those to be incidentally-learned [absent a remember cue with no prior instruction to learn (Rugg et al., 1997)]. We can imagine more than one outcome of this manipulation. First, based on the reconsolidation literature noted above, the rate of intrusions (attributing List 2 objects to the old, reactivated List 1 memory) may be lower for objects that are intentionally encoded if these are more strongly associated with the List-2 learning context compared to the more weakly learned incidentally-encoded objects. Alternately, given that forming new memories requires a sufficient level of encoding (Wichert et al., 2013), intentionally learned, but not incidentally-learned, List 2 objects may be more likely to be incorporated into List 1 memories. By identifying the factors controlling the likelihood of intrusion of new details into an existing memory, we hope to gain a more complete mechanistic understanding of the underlying reconsolidation and updating process. This knowledge, in turn, could provide clues for how to integrate memory reconsolidation into clinically therapeutic interventions for psychological disorders that involve memory components, such as post-traumatic stress disorder or specific phobias (see Lane and Nadel, 2020).

## Materials and Methods

### Subjects

A total of 85 college-age students from The University of Arizona participated in this study. We administered oral and written consent prior to participation. Our procedures were approved by the Institutional Review Board of Arizona. Subjects received course credits for their time. Subjects were randomly assigned to conditions. We eliminated 11 participants who reported failure to follow instructions by mentally rehearsing object lists in between learning sessions. A further 7 participants were outliers (greater than 2.5 standard deviations) across our dependent variables in both learning modes for correct recognition or intrusions and were thus excluded from the final analysis. Final subject counts for conditions were *n* = 35 (Reminder) and *n* = 32 (No Reminder).

### Stimuli

Our experimental stimuli consisted of 68 common objects (see Table 1). During learning and test, participants saw an image of each object in the center of a computer screen (16 in × 10.25 in) on a white background. We presented stimuli using EPrime 2.0 software (Psychological Tools, Pittsburgh, PA, United States). For List 1, we paired each object with its associated sound, e.g., participants saw a smoke detector and heard the sound of a smoke detector alarm. For List 2, participants saw 14 of the 28 objects with a blue border surrounding the image. We counterbalanced the blue borders across subjects. Between the learning of Lists 1 and 2, subjects performed one of two distractor tasks to minimize mental rehearsal, either counting diamonds on a blue background or gray birds on a white background.

**TABLE 1:** Lists of objects presented at Session 1, Session 2, and novel objects for the recognition test.

List 1	List 2	Novel objects
Airplane	Balloon	Ambulance
Alarm clock	Blow-dryer	Ball
Apple	Calculator	Bell
Arrow	Cup	Camera
Car	Drill	Cork
Coins	Eraser	Golf club
Cymbals	Feather	Gong
Door	Flashlight	Hairspray
Drum	Flower	Noisemaker
Fan	Glue	Nutcracker
Frying pan	Soda	Phone
Hands	Spoon	Pot
Leaf blower	Toothbrush	Spring
Matchstick	Whistle	Tissues
Saw	Band-aid	Train
Smoke detector	Chime	Typewriter
Sprinkler	Crayon	Vacuum
Teakettle	Dice	Washing machine
Toilet	Feather	Whip
Zipper	Hammer	
	Key	
	Sock	
	Sponge	
	Stapler	
	Sunglasses	
	Teabag	
	Tennis ball	
	Watch	

### Procedure

**FIGURE 2:**
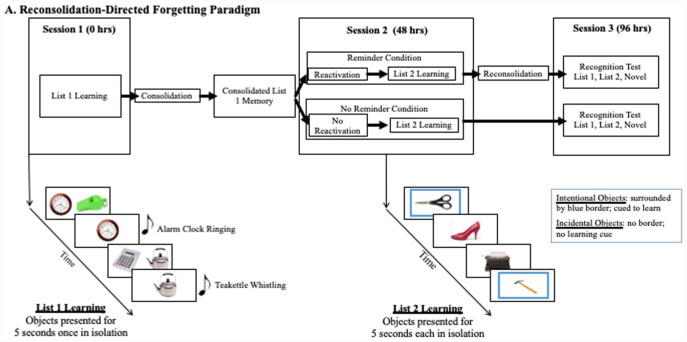
Schematic of our reconsolidation paradigm with the directed forgetting manipulation embedded in Session 2. At Session 1 subjects learn List 1 with associated sounds. Subjects are initially presented a randomized pair of objects, of which they chose which to first see in isolation where they heard the associated sound and viewed the object for 5 s. At Session 2, subjects learned novel List 2 objects. Subjects were instructed to remember the objects surrounded by a blue border (intentional-learning) but were not provided a learning direction for objects without a border (incidental-learning). Each object was presented for 5 s. At Session 3, subjects were administered a recognition test containing all List 1, List 2, and Novel objects.

We used a computerized version of the object-learning paradigm described by Hupbach et al. (2007). Subjects completed 3 sessions, two learning sessions and a testing session, each separated by 48 h (see Figure 2 for a timeline and testing schematic). In the first session participants learned List 1, comprising 20 common objects paired with their typically associated sounds. For example, participants saw an image of an apple and heard the sound of a person chewing. List 1 objects were presented in randomly generated pairs (e.g., apple and whistle) within a learning block. Participants chose the object they wished to hear first (e.g., the apple instead of the whistle). That object then appeared in isolation for 5 s with its accompanying sound. Afterward, the pair of objects returned to the screen and the subject chose the other object to experience in isolation. A new pair of objects then appeared on the screen, until subjects viewed all objects in isolation (see Figure 1). Subjects experienced the List 1 objects in three learning blocks. Pairings of Set 1 objects occurred randomly across blocks, such that no pairing repeated. In between learning blocks, subjects participated in one of the distractor tasks to minimize mental rehearsal. After subjects completed all three learning blocks they participated in a one-time recall test for the List 1 objects. At the end of Session 1, we explicitly instructed participants not to mentally rehearse the learned objects or think about the procedure between sessions.

In the second learning session, 48-h later, participants were randomly assigned to the Reminder or the No Reminder condition. In the Reminder condition, participants returned to the same room used in session one, with the same experimenter, and heard 10 of the previously learned List 1 object sounds. In the No Reminder condition, participants went to a new room, with a new experimenter, and did not hear any List 1 object sounds. All participants then learned 28 novel List 2 objects, each presented in isolation for 5 s across each of 3 learning blocks. A blue border surrounded half of the objects (intentionally-learned condition). The other half of the objects had no border (incidentally-learned condition). We counterbalanced objects by mode of learning across subjects. Participants received an instruction to remember the blue-bordered object, and no instruction for the non-bordered objects. The List 2 object-presentation order was randomized for each learning block. Subjects participated in a second distractor task in between blocks to minimize mental rehearsal. After learning, we administered a one-time recall test for the List 2 objects with the specific instruction to ‘please recall as many of the blue-bordered objects as you can.’ At the end of Session 2, we again explicitly instructed participants not to mentally rehearse the learned objects or think about the procedure between sessions.

During the test session, 48-h later, subjects participated in a recognition test of all List 1 and 2 objects plus 20 novel objects. Subjects saw each object in isolation on the screen and indicated if they previously learned the object. If so, they indicated during which session. List 2 objects not recognized as being previously learned were coded as Forgotten items. After participation, subjects completed an exit interview to determine if they followed the instructions not to mentally rehearse the objects or think about the procedure between sessions.

### Statistical Analyses

We analyzed our data using SPSS 24.0 (IBM Corp., 2016). To determine if our List 2 learning manipulation influenced subsequent recognition patterns, we planned to assess subjects’ overall response patterns using a 2 × 2 × 3 mixed ANOVA with a between subject factor of Condition (Reminder and No Reminder) and within subject factors of Learning Mode (Intentional and Incidental) and Item Type (Correct Recognition [List 2], Intrusion [List 1], or Forgotten). However, due to a violation of homogeneity of variance in the Intentional-learning condition (for Correct Recognition and Intrusions, *p*’s < 0.001), we ran the non-parametric Kruskal–Wallis test to assess differences across the 12 groups (2 Conditions [Reminder and No Reminder], 2 Learning Modes [Intentional and Incidental], and 3 Item Types [Correct Recognition [List 2], Intrusion [List 1], and Forgotten]). We then followed up with *post hoc* comparisons using Mann–Whitney tests to investigate if there was evidence to support either of our initial hypotheses: (1) stronger learning strength (for intentionally-learned objects) will show reduced intrusion rates due to increased List 2 object accuracy or (2) stronger learning strength (intentionally-learned objects) will show greater intrusion rates. Additionally, previous IMDF literature repeatedly demonstrates that to-be-forgotten information is retained at lower rates over time (Saletin et al., 2011). We thus compared forgetting rates within Condition for Intentionally-learned and Incidentally-learned objects as we expect to observe high rates of forgetting for Incidentally-learned compared to Intentionally-learned objects in both the Reminder and No-reminder conditions. This would signal that embedding the IMDF protocol into List 2 learning was successful in a reconsolidation paradigm and further provide information regarding the nature of the error rates for Incidentally-learned objects.

## Results

**FIGURE 3:**
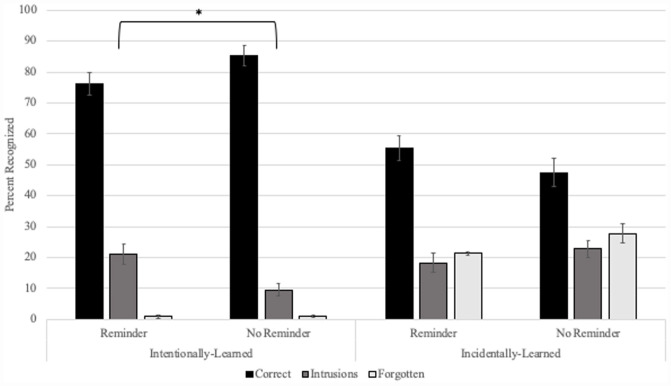
List 2 recognition by condition. Recognition pattern for List 2 objects learned intentionally or incidentally. Intentionally learned objects were recognized significantly more often as List 1 in the Reminder compared to the No-Reminder condition. Incidentally-learned objects appeared to show similar misattribution recognition rates to List 1 and high rates of forgetting regardless of condition. Asterisk denotes significance *p* < 0.05.

Figure 3 shows means by condition. The literature on memory reconsolidation provides evidence that under conditions of reactivation, new information can become linked to older memories, manifested in our experimental paradigm as intrusions of List 2 items into List 1 object memories. In tandem with this outcome, the directed-forgetting literature provides evidence that intentional learning results in more stable and detailed memories as opposed to incidental learning which results in weaker, less detailed memories that are more prone to error (Bjork and Bjork, 2003; Taylor, 2005; Fawcett and Taylor, 2010; Taylor and Fawcett, 2011; Fawcett et al., 2013). As such, our results could provide evidence to support one of two hypotheses. According to our first hypothesis, if intentional learning results in a stronger, more-detailed List-2 memory than it would be less likely to associate with List 1 in the form of intrusions. We would thus expect to observe low rates of intentionally-learned objects as intrusions, low rates of forgotten objects and high correct recognition in both the Reminder and No-Reminder conditions. At the same time, if incidentally-learned objects are weaker and more prone to error, then we should observe equally high rates of intruded and forgotten objects in both the Reminder and No-Reminder conditions combined with lower rates of correct recognition relative to intentional learning. According to our second, alternate hypothesis, a more stable intentionally-learned trace may provide critical linkage to a reactivated memory as compared to incidentally-learned objects. If so, we should observe higher rates of intrusions for intentionally-learned objects in the Reminder compared to the No-Reminder condition and low rates of forgetting in both conditions. In conjunction, if a sufficient level of encoding is necessary for intrusions, and memories for incidentally-learned objects are weaker and more prone to error, we should not observe a pattern of attributing incidentally-learned List-2 objects to List 1 only. Rather we should observe high rates of incidental object forgetting in both Reminder and No-Reminder conditions combined with lower rates of correct recognition.

### General List 2 Recognition Pattern

To determine if our List 2 learning manipulation influenced subsequent recognition patterns, we first assessed subjects’ overall response patterns for List 2 items using a 2 × 2 × 3 mixed ANOVA as described above. We observed a main effect of Item Type [*F*(2,130) = 2.27 *p* < 0.001], a two-way interaction of Learning Mode by Item Type [*F*(2,130) = 7.569, *p* < 0.001], and a three-way interaction of Condition by Learning Mode by Item Type [*F*(2,130) = 9.002, *p* < 0.001]. We report Greenhouse–Geisser corrected p-values to control for violations of sphericity. As we found a significant violation of homogeneity of variance in our Intentional-learning condition for correct recall and intrusions (*p*’s < 0.001), we used the Kruskal–Wallis test to determine if there were significant differences across the 12 groups as described above. This test showed statistically significant differences across the different categories, χ^2^(11) = 2.78.205, *p* < 0.001 allowing us to pursue planned comparisons to evaluate recognition pattern differences within Learning Modes and Conditions.

### Intrusions

We evaluated our hypotheses by conducting a Kruskal–Wallis test across the four groups (Reminder-Intentionally-learned, No Reminder-Intentionally-learned, Reminder-Incidentally-learned, and No Reminder-Incidentally-learned). Intrusion rates between the groups differed significantly [χ^2^(3) = 9.596, *p* = 0.022], with a mean rank of 71.77 for Reminder-Intentional, 50.11 for No Reminder-Intentional, 69.50 for Reminder Incidental, and 78.03 for No Reminder-Incidental objects. Therefore, we evaluated the patterns of intrusions across conditions (Reminder or No Reminder) by Learning Mode (Intentional or Incidental) using *post hoc* Mann–Whitney tests. Our competing hypotheses were that one of the two Learning Modes (Intentional or Incidental) should have higher intrusion rates in the Reminder Condition.

#### Intentional Learning

A *post hoc* Mann–Whitney *U* test comparing Reminder vs. No Reminder conditions showed significantly higher rates of intrusions in the Reminder (Mean Rank = 38.71) compared to the No-Reminder (Mean Rank = 28.84) condition [*U*(N*_*Reminder*_* = 35, *N*_No Reminder_ = 32) = 395.00, z = −2.132, *p* = 0.033].

#### Incidental Learning

A *post hoc* Mann–Whitney *U* test comparing Reminder and No Reminder conditions resulted in no significant difference in intrusion rates between the Reminder (Mean rank = 32.03) and No Reminder (Mean rank = 36.16) conditions [*U*(*N*_*Reminder*_ = 35, *N*_No Reminder_ = 32) = 629.00, *z* = 0.876, *p* = 0.381].

### Forgotten List-2 Items

Given the violations of homogeneity observed for Intrusions and Correct items and for the sake of consistency we conducted non-parametric tests on Forgotten List-2 items. We first conducted a Kruskal–Wallis test across the four groups (Reminder-Intentionally-learned, No Reminder-Intentionally-learned, Reminder-Incidentally-learned, and No Reminder-Incidentally-learned). Rates of Forgotten items between the groups differed significantly [χ^2^(3) = 86.764, *p* < 0.001] with a mean rank of 37.23 for Reminder-Intentional, 39.56 for No Reminder-Intentional, 92.49 for Reminder Incidental, and 101.22 for No Reminder-Incidental objects.

#### Intentional Learning

A *post hoc* Mann–Whitney *U* test comparing Reminder and No Reminder conditions resulted in no significant difference in rates of forgotten items between the Reminder (Mean rank = 32.94) and No Reminder (Mean rank = 35.16) conditions [*U*(*N*_*Reminder*_ = 35, *N*_No Reminder_ = 32) = 597.00, z = 0.826, *p* = 0.409].

#### Incidental Learning

A *post hoc* Mann–Whitney *U* test comparing Reminder and No Reminder conditions resulted in no significant difference in rates of forgotten items between the Reminder (Mean rank = 30.87) and No Reminder (Mean rank = 37.42) conditions [*U*(*N*_*Reminder*_ = 35, *N*_No Reminder_ = 32) = 669.5, z = 1.388, *p* = 0.165].

The presence of a significant difference among groups revealed by the Kruskal–Wallis test in combination with the absence of differences in the Mann–Whitney *U* tests supports the idea that the difference in rates of forgetting stems from differences in learning mode. This is further supported by significantly higher rates of forgetting for Incidentally-learned compared to Intentionally-learned objects in both the Reminder [Intentional mean rank = 20.77; Incidental mean rank = 50.23; *U*(*N*_*Reminder*_ = 35, *N*_No Reminder_ = 32) = 1,128.00, z = 6.528, *p* < 0.001] and No-Reminder conditions [Intentional mean rank = 18.05; Incidental mean rank = 46.95; *U*(*N*_*Reminder*_ = 35, *N*_No Reminder_ = 32) = 974.5, z = 6.542, *p* < 0.001]. Thus, we observed higher forgetting rates in both Reminder and No-Reminder conditions for incidentally-learned compared to intentionally-learned objects, reflected in the differences shown in the mean rates of forgotten List-2 items in Figure 3.

### Correct List-2 Items

Given the violation of homogeneity observed for Correct items we conducted a non-parametric test on Correct List-2 items. We first conducted a Kruskal–Wallis test across the four groups (Reminder-Intentionally-learned, No Reminder-Intentionally-learned, Reminder-Incidentally-learned, and No Reminder-Incidentally-learned). Rates of Correct items differed significantly between the groups [χ^2^(3) = 49.212, *p* < 0.001] with a mean rank of 80.84 for Reminder-Intentional, 99.28 for No Reminder-Intentional, 50.34 for Reminder Incidental, and 39.89 for No Reminder-Incidental objects.

#### Intentional Learning

A *post hoc* Mann–Whitney *U* test comparing Reminder and No Reminder conditions resulted in no significant difference in rates of Correct items between the Reminder (Mean rank = 29.8) and No Reminder (Mean rank = 38.59) conditions [*U*(*N*_*Reminder*_ = 35, *N*_No Reminder_ = 32) = 707.00, z = 1.887, *p* = 0.059].

#### Incidental Learning

A *post hoc* Mann–Whitney *U* test comparing Reminder and No Reminder conditions resulted in no significant difference in rates of Correct items between the Reminder (Mean rank = 37.36) and No Reminder (Mean rank = 30.33) conditions [*U*(*N*_*Reminder*_ = 35, *N*_No Reminder_ = 32) = 442.500, *z* = −1.482, *p* = 0.138].

The presence of a significant difference among groups revealed by the Kruskal–Wallis test in combination with the absence of differences in the Mann–Whitney *U* tests supports the idea that the difference in rates of Correct item recognition stems from differences in learning in the intentional- and incidental-encoding conditions. This is further supported by significantly higher rates of Correct List 2 recognition for Intentionally-learned compared to Incidentally-learned objects in both the Reminder (Intentional mean rank = 43.61; Incidental mean rank = 27.39; *U* = 328.50, z = −3.358, *p* = 0.001) and No-Reminder conditions (Intentional mean rank = 46.12; Incidental mean rank = 18.88; *U* = 76.000, z = −5.896, *p* < 0.001). Thus, we found the expected higher List 2 correct recognition rates in both conditions for intentionally-learned compared to incidentally-learned objects, reflected in the differences shown in the mean recognition rates of correct and forgotten List 2 items in Figure 3.

### Novel Objects

As a final check we analyzed the responses to the 20 novel, never before seen objects to assess subjects’ memory discrimination accuracy at test (i.e., subjects do not incorrectly identify new, never before seen objects as previously learned as either List 1 or List 2). We report the means for responses for both the Reminder and No Reminder conditions (Reminder: *M*_Correct_ = 92.36, *SD* = 10.95, *M*_List 1_ = 3.24, *SD* = 4.44, *M*_List 2_ = 3.71, *SD* = 8.63; No Reminder = 89.42, *SD* = 11.42, *M*_List 1_ = 4.24, *SD* = 5.46, *M*_List 2_ = 3.52, *SD* = 5.84). A 2 (Condition: Reminder, No-Reminder) × 3 [Item Type: Correct (as novel) or List 1 (incorrect) or List 2 (Incorrect)] ANOVA with Condition a between-subjects variable and Item Type a within-subject variable resulted in a main effect of Item Type [*F*(1.565,112.691) = 1168.206, *p* < 0.001] but no interactions. *Post hoc t*-tests reveal significantly greater correct recognition than incorrectly recognized as List 1 [*t*(73) = 35.219, *p* < 0.001], significantly greater correct recognition than incorrectly recognized as List 2 [*t*(73) = 40.724, *p* < 0.001] but no difference in incorrect recognition as List 1 or List 2 [*t*(73) = 1.222, *p* = 0.222]. The fact that subjects accurately detect and reject novel objects provides support for the idea that the pattern of performance in the incidental-encoding condition reflects a general pattern of memory errors as opposed to a mixture of memory updating, forgotten items, and poor memory discrimination.

In sum, our *post hoc* analyses reveal a parallel between greater intrusion rates in the Reminder compared to the No-Reminder condition for Intentionally-learned objects typical of our reconsolidation paradigm. We see a different pattern altogether for Incidentally-learned objects reflecting highly-similar patterns of errors in the Reminder and the No-reminder conditions. Taken together, the pattern of outcomes across our different conditions maps most closely to the outcomes predicted by our second hypothesis.

### List 1 Recognition

Lastly, to ensure that we did not observe bidirectional errors reflecting source errors as opposed to the unidirectional pattern reflected in memory reconsolidation, we conducted a 2 × 2 mixed ANOVA on responses to List 1 items with the between subject factor Condition (Reminder, No Reminder) and the within-subject factor Item Type (Correct, List 2 Intrusions). We found a significant main effect of Item Type [*F*(1,69) = 1505.6, *p* ≤ 0.001], but no main effect of Condition (*p* = 0.362) nor a Condition by Item Type interaction (*p* = 0.428). Consistent with prior results (Hupbach et al., 2007, 2013), *post hoc* analyses revealed significantly higher rates of correct recognition than errors (attributing List 1 items to List 2) (*p* < 0.001). Subjects showed a high rate of correct recognition for List 1 objects (*M*_Reminder_ = 16.94, *SD* = 1.97; *M*_No Reminder_ = 16.571, *SD* = 2.73) and low rates of attributing List 1 items to List 2 (*M*_Reminder_ = 1.74, *SD* = 1.72; *M*_No Reminder_ = 1.32, *SD* = 1.36).

## Discussion

We investigated how new information, learned either intentionally or incidentally, interacted with reactivated older memories. Specifically, we asked if these post-reactivation learning factors controlled the likelihood of intrusion of new details into a reactivated memory. Two potential, opposing outcomes were hypothesized: intentionally-learned objects would either be more or less likely to be intruded than incidental objects. In fact, the pattern of recognition we observed for intentional and incidental objects fits neither of these hypotheses perfectly, leading us to suggest alternative interpretations below.

### Implementation of the IMDF Manipulation

First, we successfully modified our original episodic reconsolidation paradigm (see Hupbach et al., 2007) to incorporate a modified directed-forgetting learning manipulation in the List 2 learning. Typical IMDF memory studies show higher retention rates for to-be-remembered information compared to to-be-forgotten information. As previously discussed, we used the to-be-remembered instruction to cue subjects to intentionally-learn half of the objects and no instruction for the other half of the objects, those incidentally-learned. In line with previous IMDF paradigms, in both conditions, subjects showed greater correct recognition of intentionally-learned than incidentally-learned objects. Concurrently, subjects showed greater rates of forgetting of incidentally-learned than intentionally-learned objects. Together, these findings demonstrate the success and effectiveness of our IMDF manipulation to direct subjects to use specific learning strategies, a manipulation resulting in differences in long-term memory performance.

### Mixed Evidence for Learning Manipulation

For intentionally-learned objects, subjects showed the same pattern of effects documented in our prior studies (Hupbach et al., 2007, 2009; Simon et al., 2017). Namely, in the Reminder- compared to the No-Reminder condition, there were significantly higher intrusion rates and low rates of object forgetting. However, subjects showed a different recognition pattern for incidentally-learned objects. Here, in both conditions, subjects showed equally high rates of intrusions and forgetting of objects.

This result with the incidentally-learned objects raises important questions for our understanding of updating in episodic reconsolidation. Research from our group (Hupbach et al., 2007, 2009; Nadel et al., 2012; Hupbach, 2015; Simon et al., 2017) and from others (Nader et al., 2000; Walker et al., 2003; Gershman et al., 2013), has shown that memories are vulnerable to modification only after successful reactivation (i.e., the Reminder Condition). In the absence of reactivation (i.e., the No Reminder condition), the relevant memory does not enter a labile state and remains non-modifiable. How then should we interpret the mis-attribution of incidentally-learned objects in the No-Reminder group?

One issue concerning the updating observed in episodic memory reconsolidation studies is whether it reflects actual modification of an old, reactivated memory or, instead, source discrimination errors. In general, when forming a new memory, event details are theorized to be linked to the memory origin or source (Johnson et al., 1993). When recollecting a memory at a later time, the episode is reconstructed to include its source and details. Johnson et al. (1993) reported that encoding conditions can enhance or reduce the linkage of the memory source to its details. Poor encoding would yield inadequate linkage, resulting in greater misattribution of details to alternative sources. Paralleling this, enhanced encoding would yield stronger links between the source and its details, resulting in more accuracy in the attribution of details to sources. Supporting evidence includes research on learning demands in which formal instruction alters subsequent learning behavior. Here, the change in learning demands results in increased effort, motivation and rehearsal (Naveh-Benjamin et al., 2014) and overall superior performance (Postman and Phillips, 1954). In our study, directing subjects to intentionally learn some of the objects likely affected the strength of object encoding, with incidental learning resulting in weaker encoding of the material. This could lead to reduced binding of List 2 incidental objects to their original source, and hence an increased likelihood of source errors during a later memory test.

In our paradigm, source discrimination errors could be observed in two ways. The first involves the bidirectional misattribution of objects, i.e., List 1 objects would be attributed to List 2 as often as List 2 objects were attributed to List 1. In prior work (Hupbach et al., 2009), used a recognition test to query subjects’ source evaluation of each individual List 1 and List 2 object. Subjects in the Reminder condition showed intrusions of List 2 objects into List 1 at a much higher rate than those in the No Reminder condition. Importantly, there was little evidence of source errors in either condition, defined as List 1 objects attributed to List 2. We replicated this pattern here, and did not observe List 1 objects misattributed to List 2. Instead we observed unidirectional updating for both intentional and incidental object learning in the Reminder condition which could lead one to conclude that incidentally-learned objects initiate updating at the same rate as intentionally-learned objects.

The second way source discrimination could be manifested would be as an increase in the recognition of List 2 objects as belonging to List 1 in the No Reminder condition. As noted above, prior reconsolidation literature has consistently held that memory alteration can only occur when a memory has been reactivated. In our paradigm that occurred only in the Reminder condition, where List 1 underwent reactivation, re-inducing a labile memory. Since by definition this cannot happen in the No-Reminder condition, the identification of List 2 objects as belonging to List 1 must result from something other than memory updating. The equally high intrusion rates of incidental objects in both the Reminder and No-Reminder conditions, and the equally high rates of forgetting, suggest that we observed source errors. Our results, and this interpretation, are consistent with the findings of Wichert et al. (2013), who showed that stronger encoding, through multiple presentations, increased the chances of a new item being attributed to an old, reactivated memory. In contrast, incidentally-learned objects would be less likely to form such contextual links, opening them up to source-attribution errors.

### Extension of Findings

We are thus left with two possible interpretations. One is that incidentally-learned objects are incorporated into the original memory. The other is that incidentally-learned objects do not bind strongly to the context and intrusions combined with high rates of forgetting reflect source memory errors. How might we decide between these two views? It is commonplace now to think about memory as a predictive system, and that what happens to a reactivated memory turns on how accurate its predictions prove to be (Merlo et al., 2014; Simon et al., 2017). The difference between prediction and reality is computed as a “prediction error” (PE), and the extent of PE helps determine the fate of the reactivated memory. In fear-based research, PE is easily computable with more violation resulting in the creation of a distinct and competing extinction memory and less violation resulting in reconsolidation (Merlo et al., 2014). In our previous fMRI study, activity in the TPJ appeared to reflect the extent of PE (Simon et al., 2017). Perhaps there is a prediction error account of our results. In the present study, unfortunately, it is not at all clear how prediction error and learning mode interact, hence this intriguing explanatory framework cannot be readily applied to our data.

One way to resolve these interpretations in future work would be to employ the methods used by Gershman et al. (2013) to determine whether List 2 objects that are preceded by the reinstatement of brain states associated with the original List 1 memory go on to become intrusions into List 1 memory in the No-Reminder condition. If, incidentally-learned List 2 objects showed the same pattern of reinstatement at encoding linked with later intrusions, this would suggest that weakly-encoded items can be incorporated into an earlier memory regardless of whether that memory is explicitly reactivated, an outcome at odds with the present state of reconsolidation theory. Similarly, converging evidence could be shown with a replication of the Simon et al. (2017) methodology using the different learning modes. TPJ activity could be crucial in providing converging evidence for either interpretation.

## Conclusion

Our results indicate that the type of learning subjects engage can affect memory reconsolidation processes. When and exactly how this happens remain open questions. Intentional encoding of new information can result in the updating of reactivated memories whereas memories that are not reactivated cannot be updated by new information. On the other hand, incidental encoding of new information can result in what appears to be memory updating whether an old memory was explicitly reactivated or not, an outcome that has not been observed in prior reconsolidation studies. A contrasting interpretation that might account for our pattern of results assumes that new incidental learning does yield information acquisition, but without linkage to context. This in turn leads to source errors during recognition tests, independent of the reactivation status of the older memories. Additional work is needed to help decide between these two interpretations of the data.

## Data Availability Statement

The datasets generated for this study are available on request to the corresponding author.

## Ethics Statement

The studies involving human participants were reviewed and approved by University of Arizona Human Subjects Protection Program. The patients/participants provided their written informed consent to participate in this study.

## Author Contributions

KS designed the study and collected and analyzed the data. LN and RG assisted with study design and analysis of the findings. All authors contributed to writing the manuscript.

## Conflict of Interest

The authors declare that the research was conducted in the absence of any commercial or financial relationships that could be construed as a potential conflict of interest.
